# The Portuguese version of the European Deprivation Index: Development and association with all-cause mortality

**DOI:** 10.1371/journal.pone.0208320

**Published:** 2018-12-05

**Authors:** Ana Isabel Ribeiro, Ludivine Launay, Elodie Guillaume, Guy Launoy, Henrique Barros

**Affiliations:** 1 EPIUnit–Instituto de Saúde Pública, Universidade do Porto, Porto, Portugal; 2 Departamento de Ciências da Saúde Pública e Forenses e Educação Médica, Faculdade de Medicina, Universidade do Porto, Porto, Portugal; 3 U1086 INSERM UCN "Anticipe", Caen, France; University Complutense of Madrid, SPAIN

## Abstract

Socioeconomic inequalities are major health determinants. To monitor and understand them at local level, ecological indexes of socioeconomic deprivation constitute essential tools. In this study, we describe the development of the updated version of the European Deprivation Index for Portuguese small-areas (EDI-PT), describe its spatial distribution and evaluate its association with a general health indicator–all-cause mortality in the period 2009–2012. Using data from the 2011 European Union–Statistics on Income and Living Conditions Survey (EU-SILC), we obtained an indicator of individual deprivation. After identifying variables that were common to both the EU-SILC and the census, we used the indicator of individual deprivation to test if these variables were associated with individual-level deprivation, and to compute weights. Accordingly, eight variables were included. The EDI-PT was produced for the smallest area unit possible (n = 18084 census block groups, mean/area = 584 inhabitants) and resulted from the weighted sum of the eight selected variables. It was then categorized into quintiles (Q1-least deprived to Q5-most deprived). To estimate the association with mortality we fitted Bayesian spatial models. The EDI-PT was unevenly distributed across Portugal–most deprived areas concentrated in the South and in the inner North and Centre of the country, and the least deprived in the coastal North and Centre. The EDI-PT was positively and significantly associated with overall mortality, and this relation followed a rather clear dose-response relation of increasing mortality as deprivation increases (Relative Risk Q2 = 1.012, 95% Credible Interval 0.991–1.033; Q3 = 1.026, 1.004–1.048; Q4 = 1.053, 1.029–1.077; Q5 = 1.068, 1.042–1.095). Summing up, we updated the index of socioeconomic deprivation for Portuguese small-areas, and we showed that the EDI-PT constitutes a sensitive measure to capture health inequalities, since it was consistently associated with a key measure of population health/development, all-cause mortality. We strongly believe this updated version will be widely employed by social and medical researchers and regional planners.

## Introduction

Poor socioeconomic circumstances are one of the strongest predictors of morbidity and mortality worldwide and might be modifiable by policies at the local, national, and international levels [1]. Therefore, tackling social inequalities in health is a major priority [2].

Small-area measures of socioeconomic deprivation are important tools for quantifying multidimensional social and material disadvantage and for studying health inequalities [3]. Because they are produced for small-areas, composed of few inhabitants, these indexes minimize the risk of ecological bias (i.e. the difference between estimated associations based on ecological and individual-level data) [4]. Moreover, ecological indexes of socioeconomic deprivation constitute tools that support decision-making processes aimed at the improvement of people’s health and well-being [5].

In 2016, a multinational and multidisciplinary team joined efforts to create a cross-national ecological deprivation index for the small areas of England, France, Italy, Portugal, and Spain—the European Deprivation Index (EDI) [6]. This measure filled up an important methodological gap and constituted the first index of socioeconomic deprivation for Portuguese small-areas [7]. The EDI was built using data from the 2001 census and the 2006 EU-SILC survey (European Union-Statistics on Income and Living Conditions) and it was grounded on a solid theoretical framework, individual and aggregated variables, and on an annual Europe-wide survey allowing its replication over the time and in any European country.

Since then, multiple studies, at international and national-level, have used the EDI to investigate inequalities in health outcomes, namely longevity and survival [8, 9], hospital death [10], cancer [11–13], infections [14] and hip fracture [15]. Most studies showed that the more deprived areas presented poorer health outcomes as compared to the less deprived and some concluded that this indicator alone explained a considerable amount of the observed between-area differences in the studied outcomes [8, 9]. This index has also been recently used to explore problems of socioeconomic inequalities in the distribution of detrimental physical exposures–the so-called environmental injustice–which were found to be concentrated in more deprived areas [16, 17].

Social and economic changes, particularly evident in the last decade due to the recent economic recession [18, 19], justified the need to update the EDI using more recent surveys and census data. The concept of deprivation is time- and context-specific [20, 21] and there is evidence that economic recessions generate new pockets of poverty widening social inequalities [22]. Therefore, we describe the development of an updated version of the European Deprivation Index for Portuguese small-areas (EDI-PT), describe its distribution across the Portuguese territory, and evaluate its association with a general health indicator–all-cause mortality.

## Materials and methods

### Data sources

To obtain individual-level data on deprivation, we used the European Union-Statistics on Income and Living Conditions survey, EU-SILC [23]. EU-SILC is being implemented by the European Statistical System (ESS) since 2004, to measure deprivation in several domains (income, social exclusion, housing conditions, labour, education and health). In Portugal, EU-SILC is conducted annually since 2006 by INE, Instituto Nacional de Estatística (Statistics Portugal). We used the 2011 EU-SILC cross-sectional survey which covered all 28 EU countries (plus Iceland, Norway, Switzerland and Turkey). In Portugal this survey took place between May and August 2011 and included 5740 households and 12 489 individuals aged 16 years old or more.

Ecological data on deprivation came from the 2011 Portuguese Population and Housing Census available from INE [24], which was the latest census organized in Portugal. In Portugal, censuses are universal and exhaustive, covering the entire population. Although data was available at census block level, we opted to build EDI-PT at an upper aggregation level, census block groups, because: 1) a considerable proportion (9%) of the census blocks had zero residents, 2) we aimed to guarantee comparability with the previous version of the EDI, and 3) census block groups are still amongst the smallest geographical units used in the countries for which the EDI has been constructed and, for this reason, issues such as ecological bias are unlikely to be a problem In 2011, there were 10 562 178 inhabitants, 18 074 census block groups, each one with 221 households and 584 inhabitants on average.

To examine the association between the EDI-PT and mortality, we retrieved data on the number of deaths and inhabitants according to age group, sex and parish in Portugal for the period 2009–2012, before the 2013 administrative reorganization, when the Portuguese parishes were aggregated and some inter-parish borders were modified, leading a 27% decrease in the number of parishes. The process of aggregation reduces information, increases within-area heterogeneity and may prevent us to detect important inequalities and associations [25]. We used a 4-year period, instead of a single year, to avoid the well-known small number problems. Portuguese parishes can have very few inhabitants (population range: 31–66 250) and many have less than 100 inhabitants. Small populations tend to give rise to the most extreme event rates. Aggregating data in time is a common procedure in spatial statistics to efficiently deal with this problem [4].

### EDI-PT construction

The construction of the EDI-PT comprised three steps, similarly to the previous version of the EDI: (1) construction of an individual deprivation indicator; (2) identification of the variables that were available at individual (survey) and at aggregate level (census) and (3) construction of an ecological deprivation index, the EDI-PT.

#### Step 1: Construction of an individual deprivation indicator

Firstly, we constructed an individual deprivation indicator based exclusively on EU-SILC data, which involved the following tasks:

- **Selection of fundamental needs:** Fundamental needs are items that are considered necessary in a specific sociocultural context, and possessed by the majority of the population, so that those that cannot afford it are considered in disadvantage. Only items possessed by more than 50% of the households were considered fundamental needs among the nine items representing material deprivation in the EU-SILC Survey.
- **Identification of fundamental needs associated with objective and subjective poverty:** Individual deprivation is closely related to poverty. To identify the previously identified fundamental needs that were associated with poverty, we restricted to those associated simultaneously with objective (income) and subjective (perceived) poverty, both measured in EU-SILC survey. In objective terms, an individual is considered at-risk-of-poverty if his/ her household income is below 60% of the national median equivalised disposable income, i. e., the total income of a household, after tax and other deductions, that is available for spending or saving, divided by the number of household members converted into equalised adults [26]. In Portugal, the threshold was 5046 euros per year, in 2011. Based on that, 18.0% of the households were considered poor [27]. Subjective poverty was evaluated by the EU-SILC Likert-scale question ‘ability to make ends meet’ (from 1—with great difficulty to 6—very easily). To determine the threshold at which a person felt poor, we carried out univariable logistic regressions between objective poverty (‘poor’/’not poor’, based on the 5046 euros threshold) and subjective poverty, dichotomized according to all combinations of answers to the question ‘ability to make ends meet’. Wald chi-square statistic (χ^2^) was used to determine the dichotomization with the best fit; the higher χ^2^ the better. The answer 1 (‘with great difficulty’) versus the others (2–6) had the highest χ^2^. Based on that threshold, 19.2% of the Portuguese households were subjectively poor. From the fundamental needs identified in (a), only those significantly associated with both subjective and objective poverty were selected for the next step. Univariable and multivariable logistic regression models were run to identify them with a significance level of 5%.
- **Creation of an individual deprivation indicator:** Subsequently, the above mentioned fundamental needs were utilized to create a binary indicator of individual deprivation. Multivariable logistic regression (assessed by Wald χ^2^ statistic) was fitted to determine the threshold number of fundamental needs that better explained both objective and subjective poverty. This threshold was used to classify individuals as deprived or not.

#### Step 2: Identification and arrangement of the variables that were available at individual level (EU-SILC survey) and at ecological level (census)

The second phase in the creation of EDI involved both individual and ecological data. First, we assessed which variables of the EU-SILC survey were also present in the 2011 Portuguese Census data. Then, we recoded the variables in both datasets (EU-SILC and Census) so that they become comparable. In order to calculate proportions at ecological level, we had to dichotomize all the variables that could assume more than two values. For that, univariable logistic regression models were run between the individual deprivation indicator and the variables present in both Census and EU-SILC dichotomized in all possible ways. We selected the dichotomization which yield the best model fit (highest Wald χ^2^).

#### Step 3: Construction of an ecological deprivation index, EDI-PT

To determine which pre-selected variables were to include in the EDI-PT, a multivariable logistic regression was run and only variables significantly associated with the individual deprivation indicator were kept. The regression coefficients of that model became the weights assigned to each of these variables, after they were normalized to the national mean (z-scores). The EDI-PT resulted from the weighted sum of the normalized variables. Finally, the census block groups were categorized into quintiles (Q1-least deprived to Q5-most deprived) to facilitate interpretation.

### Associations with all-cause mortality

To estimate associations with all-cause mortality we used a hierarchical Bayesian spatial model. The Bayesian inference combines the prior distribution on model parameters and the data likelihood to derive the posterior distribution. The main advantage of the Bayesian approach resides in its taking into account uncertainty in the estimates and its flexibility and capacity of dealing with issues, such as spatial autocorrelation and large variance of small areas [28]. Besides, to guarantee that the associations were not driven by the different age structures of the Portuguese parishes, mortality rates were age-standardized using the indirect method.

We used the Portuguese mortality rates by sex and age group (5-year age groups) as reference to compute the expected number of deaths.

We assumed that the response variable, deaths (*O*_*i*_), in each *i*_*th*_ area follows a Poisson distribution, where *E*_*i*_ is the expected number of deaths and *θ*_*i*_ the relative risk (RR) (Eq 1):

$$O_{i}∼Poisson(E_{i};\;\theta_{i})$$ (Eq 1)

$$log(\theta_{i})=\alpha +\beta_{xi}+s_{i}$$ (Eq 1.1)

where *α* is an intercept quantifying the average number of deaths in the 4050 parishes, and *β*_*xi*_ the effect of the socioeconomic deprivation. The area-specific effect *s*_*i*_ was modelled based on a Besag, York and Mollie (BYM) model [29], with the parameterization suggested by Dean and co-authors [30]. (Eq 1.2)

$$s_{i}=\tau \;(\sqrt{\varphi *u_{i}}+\sqrt{1-\varphi *v_{i}})$$ (Eq 1.2)

where *u*_*i*_ is the structured effect and *v*_*i*_ the unstructured effect. The *u*_*i*_ effect was scaled to make the model more intuitive and interpretable [31], so that *u* expresses the proportion of the spatial effect due to the structured part and 1/*s* is the marginal variance of *s*_*i*_. We used a adjacency-based criteria to create the spatial weights matrix, except for isolated geographical areas (islands) where we used a distance-based criteria, i.e., if an area was isolated and had no adjacent areas we consider as neighbor the geographical area located at the closest distance.

Associations were expressed in RRs, which denote the ratio between the risk of death of a deprivation quintile and the risk of the reference quintile (the least deprived, quintile 1, was used as the reference). An RR would be considered significantly higher or lower if its 95% credible intervals (95%CrIs) did not include the value 1. RRs and 95%CrIs were derived from their posterior means and quintiles. Posterior distributions were obtained using the Integrated Nested Laplace Approximation (INLA), which was implemented in the R-INLA library [32].

#### Sensitivity analysis

To assess the potential impact of the MAUP (Modifiable Areal Unit Problem), the same hierarchical Bayesian spatial model was fitted using municipalities (n = 308), an upper level geography, as geographical unit of analysis.

## Results

### Construction of an individual deprivation indicator

#### Identification of fundamental needs

Table 1 lists the items considered fundamental needs in Portugal in 2011. Their lack reflects deprivation. From the nine items assessed in the Portuguese EU-SILC survey, taking a week’s annual holiday away from home was the only item to be excluded, as 60% of the Portuguese could not afford it.

**Table 1:** Identification of fundamental needs: Proportion of Portuguese households that indicated that specific goods and services were not within their means (EU-SILC survey 2011, n = 5740 households).

Type of need	% of households that cannot afford
Using your own means to cover a necessary yet unplanned expense	29.7
Keeping your house adequately warm	26.9
Having a personal car	9.7
Having a computer	8.8
Eating a meal containing meat, fish, or the vegetarian equivalent once every two days	3.5
Having a phone (including mobile phone)	2.8
Having a washing machine	2.5
Having a colour TV	0.7

#### Identification of fundamental needs associated with objective and subjective poverty

Six of the items of Table 1 were selected as fundamental needs: ‘Eating a meal containing meat, fish, or the vegetarian equivalent once every two days’; ‘Using your own means to cover a necessary yet unplanned expense’; ‘Keeping your house adequately warm’; ‘Having a phone (including mobile phone)’; ‘Having a washing machine’; and ‘Having a personal car’. These were the items that were significantly associated with both objective (income) and subjective deprivation (ability to make ends meet).

#### Creation of an individual deprivation indicator

The better threshold of fundamental needs that explained both objective and subjective poverty stayed on two fundamental needs, meaning that an individual that could not afford two or more (of the six) fundamental needs was defined as deprived.

### Identification and arrangement of the variables that were available at individual level (EU-SILC survey) and at ecological level (census)

We found a total of nine matching variables available in the EU-SILC survey and in the census: home ownership (renter, owner, other); presence/absence of indoor flushing in the households; presence/absence of bath/shower in the households; rooms in the household (≤5 rooms or ≥6 rooms), also employed in previous EDI [6, 7] as a proxy measure of ‘overcrowding’ (since more robust measures overcrowding were unavailable at small-area level), and this dichotomization was the one that yield the best model fit (highest Wald χ^2^) in step 2; occupation class of the residents (lower white collars, upper white collars and blue collars); education level of the residents (primary, secondary or tertiary); employment condition (employee or employer); employment status (unemployed looking for a job and employed); nationality (Portuguese or foreign).

After identifying the best dichotomization, for all variables and for each census block group, proportions were calculated as follows: percentage of non-owned households; households without indoor flushing; households without bath or shower; households with five rooms or less (pantries, kitchens, corridors, bathrooms and balconies excluded); individuals with blue-collar (i.e., manual) occupations; individuals with low education level (≤ 6th grade); non-employers; unemployed looking for a job; and foreign residents.

### Construction of an ecological deprivation index, EDI-PT

Tables 2 and 3 show the variables selected for the ecological deprivation index, which were the variables associated with the binary individual deprivation indicator. The variable “% of households without bath/shower” was removed since it was not associated with individual deprivation in the multivariable model. The regression coefficients (β) of this model were used as weights.

**Table 2:** Final model of multivariable logistic regression selecting components of EDI, which were associated with the final individual deprivation indicator, Portuguese EU-SILC (n = 12 489).

Variable	β	OR (95% CI)*
Non-owned households	1.191	3.291 (2.822–3.839)
Households without indoor flushing	1.729	5.637 (3.153–10.078)
Households with 5 rooms or less	0.964	2.621 (2.043–3.363)
Blue-collars	0.370	1.447 (1.233–1.698)
Residents with low education level (≤6 years)	0.511	1.667 (1.405–1.978)
Non-employers	0.620	1.858 (1.181–2.923)
Unemployed looking for a job	0.268	1.307 (1.127–1.516)
Foreign residents	1.038	2.823 (1.748–4.560)

*Odds ratio and 95% confidence intervals.

**Table 3:** Summary statistics of the census variables included in the construction of the EDI-PT score (n = 10 562 178 residents, n = 3 997 724 households).

Census variable	Percentage
Non-owned households	26.8%
Households without indoor flushing	0.9%
Households with 5 rooms or less	73.3%
Blue-collars	37.3%
Residents with low education level (≤6 years)	47.9%
Non-employers	89.5%
Unemployed looking for a job	13.2%
Foreign residents	3.4%

The values of the EDI-PT score for each census block group were obtained using the following equation (Eq 2), a weighted sum of the eight selected variables after normalization to the national mean (z-score):

$$\begin{array}{l} EDI-PT\;score=\\ 1.191\times \%Non-owned\;households+1.729\times \\ \%Households\;without\;indoor\;flushing+0.964\times \\ \%Household\;with\;5\;rooms\;or\;less+0.370\times \%Blue-collars+0.511\times \\ \%Residents\;with\;low\;education\;level+0.620\times \%Non-employers+0.268\times \\ \%Unemployed\;looking\;for\;a\;job+1.038\times \%Foreign\;residents \end{array}$$ (Eq 2)

### EDI-PT descriptive statistics and geographic distribution

The EDI-PT had the following distribution: minimum = -10.804; maximum = 45.484; mean = 0.000 and standard deviation = 3.149. Then, each census block group was categorized according to its level of deprivation using the quintiles of the EDI-PT score as cut-offs: 1 (-10.804 to -2.393); 2 (-2.393 to -1.019); 3 (-1.019 to 0.259); 4 (0.259 to 2.070) and 5 (2.070 to 45.484). The first quintile (least deprived) totalized 2 185 289 inhabitants (20.7% of the national population); the second, 2 199 410 (20.8%); the third, 2 189 526 (20.7%); the fourth, 2 097 658 (19.9%); and the fifth (most deprived), 1 890 244 (17.9%).

**Fig 1:**
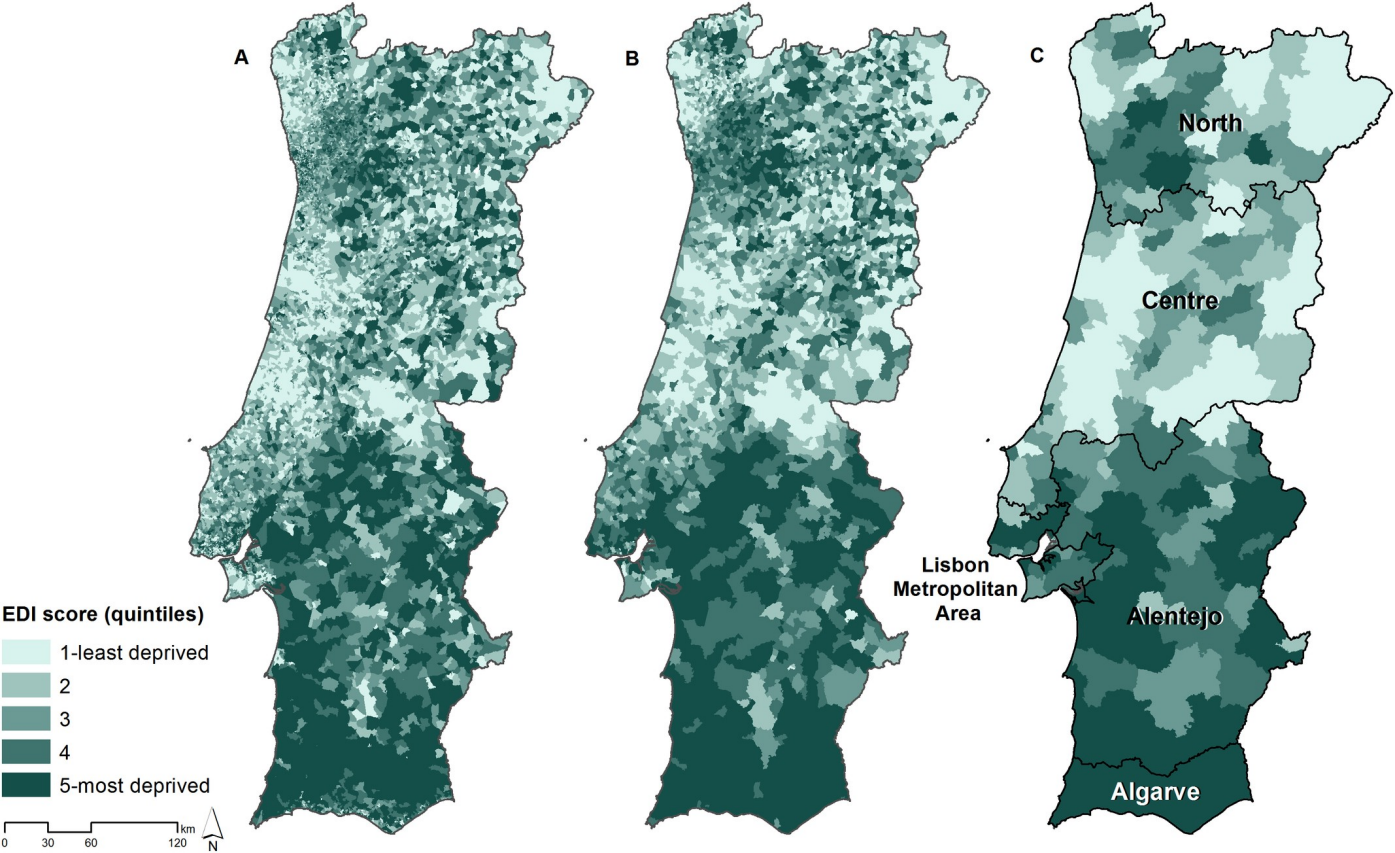
Spatial distribution of the European Deprivation Index for Portuguese small-areas in Continental Portugal. (A: Census block groups; B: Parishes; C: Municipalities).

**Fig 2:**
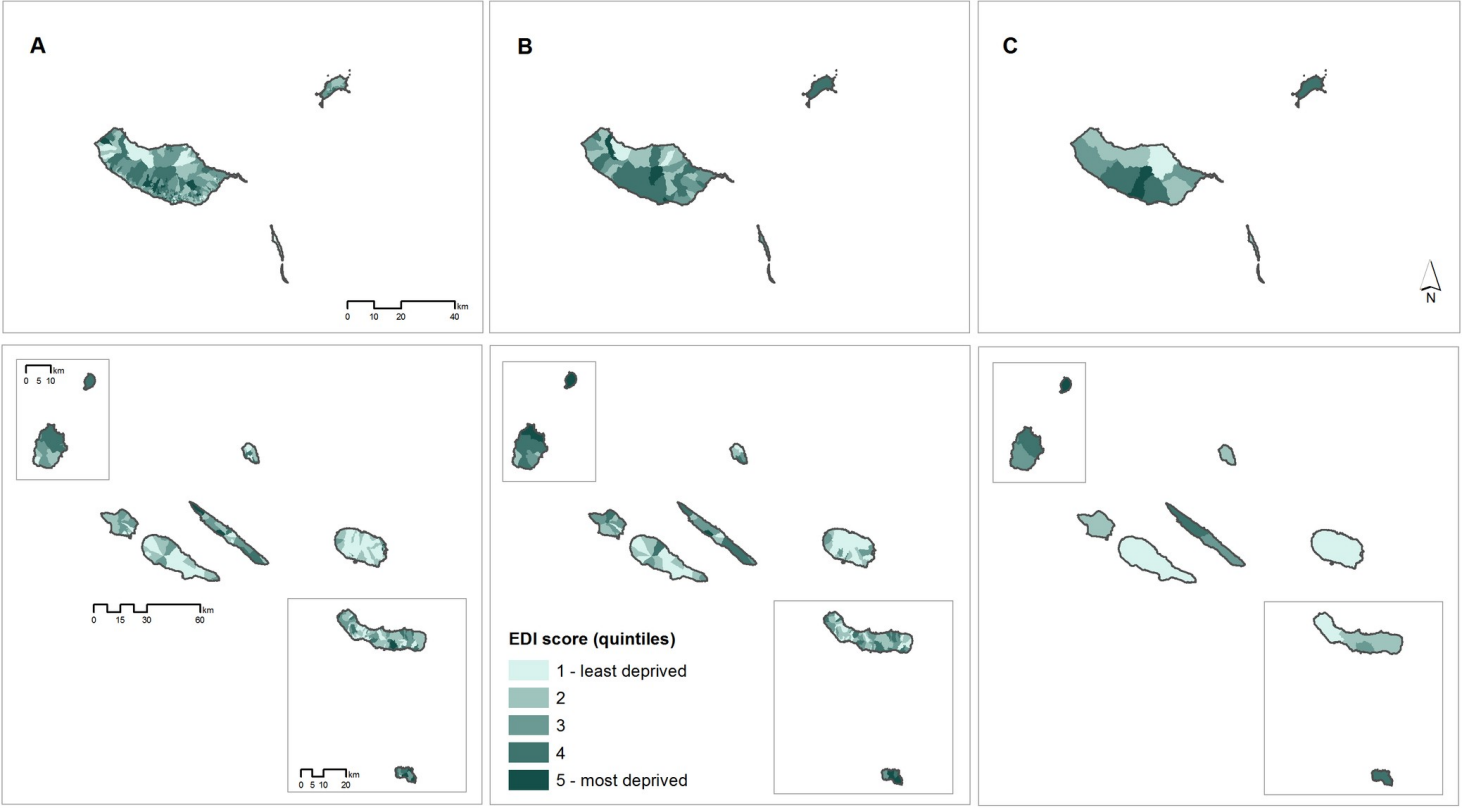
Spatial distribution of the European Deprivation Index for Portuguese small-areas in archipelagos. (A: Census block groups; B: Parishes; C: Municipalities).

The EDI-PT was also computed at higher aggregation level–parish level (n = 4260) and municipality level (n = 308). Figs 1 and 2 show the geographical distribution of the EDI in Portugal and archipelagos. It has a clear geographic pattern, being the most deprived areas generally located in the South, whereas the least deprived areas were predominantly located in the Centre and North regions.

### Association between EDI and mortality

From 2009 to 2012 there were 420 781 deaths. Posterior means of the SMRs (standardized mortality ratios) ranged from 65.6 to 294.9. Although the SMR did not show a much demarked geographical pattern, highest SMR were generally concentrated in Alentejo, archipelagos and Vila Real district and the lowest in Centre and coastal areas.

The EDI-PT was positively and significantly associated with overall mortality. As shown in Table 4, compared with the least deprived parishes, parishes in the second, third, fourth and fifth quintile of socioeconomic deprivation presented RRs of 1.012 (95%CrI 0.991–1.033), 1.026 (1.004–1.048), 1.053 (1.029–1.077) and 1.068 (1.042–1.095), respectively. It is important to refer, though, that parishes classified in the second quintile of deprivation did not register significantly higher mortality as compared with the least deprived.

**Table 4:** Association (Relative Risk and 95% Credible Intervals) between the European Deprivation Index quintiles (Q1-least deprived to Q5-most deprived) and age-adjusted mortality rates in Portugal (n = 4260 parishes, 2009–2012).

Socioeconomic deprivation	RR (95% Credible Interval)
Q1 –least deprived	1.000 (Ref)
Q2	1.012 (0.991–1.033)
Q3	1.026 (1.004–1.048)
Q4	1.053 (1.029–1.077)
Q5 –most deprived	1.068 (1.042–1.095)

Associations remained mostly unchanged when using municipalities (an upper-level geography) as unit of analysis, as shown in S1 Table.

## Discussion

In this paper, we aimed to fully describe the steps involved in the creation of the 2011 version of the European Deprivation Index for Portuguese small-areas, EDI-PT. We also demonstrated the link between the EDI-PT and a key indicator of health and development, the mortality rates, across the more than 4000 parishes of Portugal. In this analysis, we showed that mortality rates are positively associated with socioeconomic deprivation, increasing in a graded manner with increasing deprivation.

The link between mortality and deprivation is probably one of the oldest and more consistent epidemiological findings. Although Portugal is a rather small and homogeneous country, we found a gradation in the risk of death according to the socioeconomic characteristics of the areas. Similar findings have been reported all over Europe [33, 34]. It is important to refer, though, that the magnitude of the geographical differences between areas, and consequently the magnitude of the associations, is moderate when compared to other European countries; for instance, we observed that parishes in the second quintile of deprivation did not register significantly higher mortality that the least deprived. Although due to methodological reasons our results are not fully compared with the published literature, this is in accordance to other investigations that showed that the effect magnitude of socioeconomic deprivation in all-cause and cause specific mortality is smaller in southern Europe than in northern, western and central-eastern European settings [35–38]. Using the previous EDI (2001), the magnitude of the socioeconomic inequalities in old-age survival across five European countries (Portugal, England, France, Spain and Italy) was recently compared and these were found to be narrower in Portugal [39]. One the other hand, the size of the units used in this study (discussed ahead) can also explain the relative mild effect of deprivation, since the larger the areas the lesser the ability to capture inequalities.

Although comparisons need to be made with caution, when we compare the 2011 version of the EDI-PT with the previous 2001 version and, although ten years had gone by, we observed that the patterns of deprivation have remained rather stable in the last decade, being the most deprived areas generally located in the South of the country, whereas the least deprived areas were predominantly located in the Centre and North regions, even though the differences between inner and coastal areas were somewhat attenuated. This persistence in the spatial pattern of deprivation has been observed elsewhere. For instance in the UK, in London, the maps of poverty in 1800s, 1900s and 1990s are quite similar and these patterns match the distribution of key health outcomes such as mortality [40].

Although in both 2001 and 2011 versions of the EDI-PT we used eight weighted variables, it is important to refer that these do not fully overlap. For instance, in the present version of the EDI-PT, we included variables related with nationality (proportion of foreign residents) and employment condition (proportion of non-employees), whereas in the 2001 version these were absent and we included variables related with demography (age and sex) and the presence of a shower/bath in the household instead. These differences may happen due to three main reasons: 1) some variables were no longer significantly associated with deprivation (presence of bath/shower in the household); 2) methodological decisions lead to the exclusion of variables to avoid problem of over-adjustment in epidemiological studies (age and sex); and 3) changes in the questionnaires and in the data access policies lead to the inclusion of additional variables related to deprivation (nationality and employment condition). The weights attributed to each variable also suffered slight changes, showing that deprivation is a mutable, time- and context-specific state. One of the variables whose weighting changed the most was the education attainment–the magnitude of the association with deprivation was reduced from 3.640 to 1.667. The widespread improvement in national education levels might explain this reduction in the magnitude of the association.

It is also worth mention that the fundamental needs have also changed from 2001 and 2011. Fundamental needs are items considered necessary in a specific sociocultural context, and possessed by the majority of the population, so that those that cannot afford them are considered in disadvantage. In the 2001 version of the EDI-PT, having a computer was not considered a fundamental need since more than 50% of the population could not afford it; ten years later, in 2011, only 8.8% of the population could not afford a computer and it is now considered a fundamental need. Despite being considered fundamental needs both in 2001 and in 2011, we observed an increase in the proportion of households that could not cover an unplanned expense; this proportion was 18.2% in 2001 and rose to 29.7% in 2011. The 2000s economic recession that peaked around 2010 in Portugal [19], and that lead to higher unemployment rates and less social and financial benefits, may explain this substantial increase.

Naturally, the EDI-PT presents some limitations discussed in previous publications. First, the choice of the variables depends greatly on their availability, both in the EU-SILC survey and in the census, and that can obviously affect the quality of the indicator. Another important limitation is due to a widely discussed and controversial issue, the ability of a single deprivation index to discriminate between rural and urban deprivation [41, 42]. As the EU-SILC survey does not allow weighting on urban and rural areas, we could not overcome that potential limitation. Yet, we have reasons to believe that the rural/urban bias may not be a problem in Portugal. In a parallel analysis, we examined the percent distribution of each of the variables that compose the EDI according to the level of urbanity of the Portuguese parishes [43] and we observed no clear trend. As shown in S2 Table, some variables (% of households without indoor flushing, blue-collar workers and less educated people) are more common in the rural areas, while others (% non-owned and small size households, unemployed and foreign residents) are more common in the urban ones. Finally, as any type of aggregated measure, the EDI and investigations derived from may be affected by the Modifiable Areal Unit Problem, which happens when the way the data is aggregated, either in terms of scale and/or boundary delineation, affects the study conclusions, namely geographical patterns, the level of inequality and the magnitude of the associations [44]. Still, and although it is recommend the use of the smallest geographic units possible–since the use of large geographical areas can “wash away” (gerrymander) differences in covariates and outcomes–we conducted a sensitivity analysis to test the impact of using an upper-level geography (municipalities) on the associations between the EDI and mortality; we found that associations remained mostly unchanged after using these larger geographical units.

In conclusion, we showed that it is possible to build and to provide an updated index of socioeconomic deprivation for Portuguese small-areas, and, most importantly, it constitutes a sensitive measure to capture health inequalities, since it was consistently associated with a key measure of population health and development, all-cause mortality. Based on our previous experience with the EDI-PT 2001, we strongly believe this updated version will be widely employed by social and medical researchers but also by regional planners, with the ultimate goal of better understanding the health inequalities not only in Portugal but also across Europe.

## Supporting information

S1 Table Association (Relative Risk and 95% Credible Intervals) between the European Deprivation Index quintiles (Q1-least deprived to Q5-most deprived) and age-adjusted mortality rates in Portugal (n = 308 municipalities, 2009–2012). (DOCX)

S2 Table Summary statistics of the census variables included in the construction of the EDI-PT score according to parish urbanity level (n = 10 562 178 residents, n = 3 997 724 households). (DOCX)
