# Supplementary material for: The Portuguese version of the European Deprivation Index: Development and association with all-cause mortality
Source: PLoS One. 2018 Dec 5;13(12):e0208320. doi: 10.1371/journal.pone.0208320 (PMC6281298; doi:10.1371/journal.pone.0208320)
Supplement: S1 Table — (DOCX) [file pone.0208320.s001.docx]

**Association (Relative Risk and 95% Credible Intervals) between the European Deprivation Index quintiles (Q1-least deprived to Q5-most deprived) and age-adjusted mortality rates in Portugal (n=308 municipalities, 2009-2012).**

| **Socioeconomic deprivation** | **RR (95% Credible Interval)** |
| --- | --- |
| Q1 – least deprived | 1.000 (Ref) |
| Q2 | 1.029 (1.001-1.058) |
| Q3 | 1.041 (1.011-1.072) |
| Q4 | 1.052 (1.020-1.085) |
| Q5 – most deprived | 1.069 (1.032-1.107) |
