# Supplementary material for: The Portuguese version of the European Deprivation Index: Development and association with all-cause mortality
Source: PLoS One. 2018 Dec 5;13(12):e0208320. doi: 10.1371/journal.pone.0208320 (PMC6281298; doi:10.1371/journal.pone.0208320)
Supplement: S2 Table — (DOCX) [file pone.0208320.s002.docx]

**Summary statistics of the census variables included in the construction of the EDI-PT score according to parish urbanity level (n=10 562 178 residents, n= 3 997 724 households).**

| **Census variable employed in calculation**  **of the EDI-PT score** | **National** | **APR** | **AMU** | **APU** |
| --- | --- | --- | --- | --- |
| Non-owned households | 26.8% | 12.9% | 18.9% | 31.1% |
| Households without indoor flushing | 0.9% | 2.4% | 1.1% | 0.5% |
| Households with 5 rooms or less | 73.3% | 64.3% | 64.1% | 77.0% |
| Blue-collars | 37.3% | 54.1% | 49.7% | 32.1% |
| Residents with low education level (≤6 years) | 47.9% | 62.1% | 56.4% | 43.6% |
| Non-employers | 89.5% | 89.1% | 89.1% | 89.6% |
| Unemployed looking for a job | 13.2% | 12.2% | 12.0% | 13.6% |
| Foreign residents | 3.4% | 1.8% | 1.8% | 4.1% |

APR = Predominantly rural areas; AMU = Moderately urban areas; APU = Predominantly urban areas.
